# De-identifying Swedish clinical text - refinement of a gold standard and experiments with Conditional random fields

**DOI:** 10.1186/2041-1480-1-6

**Published:** 2010-04-12

**Authors:** Hercules Dalianis, Sumithra Velupillai

**Affiliations:** 1 Department of Computer and Systems Sciences, (DSV), Stockholm University Forum 100, 164 40 Kista, Sweden

## Abstract

**Background:**

In order to perform research on the information contained in Electronic Patient Records (EPRs), access to the data itself is needed. This is often very difficult due to confidentiality regulations. The data sets need to be fully de-identified before they can be distributed to researchers. De-identification is a difficult task where the definitions of annotation classes are not self-evident.

**Results:**

We present work on the creation of two refined variants of a manually annotated Gold standard for de-identification, one created automatically, and one created through discussions among the annotators. The data is a subset from the Stockholm EPR Corpus, a data set available within our research group. These are used for the training and evaluation of an automatic system based on the Conditional Random Fields algorithm. Evaluating with four-fold cross-validation on sets of around 4-6 000 annotation instances, we obtained very promising results for both Gold Standards: F-score around 0.80 for a number of experiments, with higher results for certain annotation classes. Moreover, 49 false positives that were verified true positives were found by the system but missed by the annotators.

**Conclusions:**

Our intention is to make this Gold standard, The Stockholm EPR PHI Corpus, available to other research groups in the future. Despite being slightly more time-consuming we believe the manual consensus gold standard is the most valuable for further research. We also propose a set of annotation classes to be used for similar de-identification tasks.

## Background

Health related texts and specifically Electronic Patient Records (EPRs) are an abundant source of valuable information for both clinicians, computer scientists and linguists. Text mining tools, for instance, could be developed by computer scientists for the exploration of such information rich resources. Clinicians could use these text mining tools both on individual patient cases as well as on whole EPR corpora, to find previously unknown information. Moreover, linguists could use such resources to make interesting stylistic and empirical analyses on EPR language.

We have access to a very large EPR corpus, the Stockholm EPR Corpus, containing clinical texts written in Swedish [1]. The Stockholm EPR Corpus contains over one million patient records from over 2 000 clinics. We strive to make this corpus available for a larger research community encompassing researchers in both computational linguistics and medical informatics as well as to practicing clinicians.

In order to develop methods that exploit the vast amount of information contained in EPRs, researchers need to be able to access the data itself. This is often difficult, as such data sources are often restricted due to confidentiality reasons and the like. EPR corpora contain information that can reveal the identity of the patients and hence sensitive information about the individual patient. To remove the information that can identify the individual patient one needs to de-identify the patient records.

De-identification is an extremely important and difficult task, and many questions arise. What constitutes identifiable information? How much information can be removed (or replaced), ensuring patient integrity and still keeping important information? Moreover, manually de-identifying large resources such as the Stockholm EPR Corpus in its entirety is not feasible, therefore automatic methods are needed. Still for the evaluation and training of automatic systems, manually annotated Gold standards are needed. One issue that arises is how large training set does a trainable system require in order to obtain high results? Furthermore, an interesting question to analyse is whether the merging of conceptually similar annotation classes will increase results.

In this paper we describe work on de-identification of Swedish EPRs. We have two aims: (1) refining an existing manually annotated Gold standard for de-identification purposes, one automatically refined and the other (semi-)manually refined, and (2) initiating experiments on using these refinements to evaluate an automatic machine learning system based on the Conditional Random Fields (CRF) algorithm. We have analysed the annotation classes used for de-identification and identified issues that are challenging and need further refinement.

### Related research

Using manually annotated resources for Natural Language Processing (NLP) and Information Access (IA) research is very common. Such resources are useful for at least two purposes; for empirical studies on the topic the annotations cover, and for developing and evaluating computational models. It is, however, time-consuming and costly to create such resources. Moreover, for the resources to be useful in an automated system, the annotations must be well-defined and reliable. For an annotated resource to be considered reliable, one must ensure that the annotations have high agreement among the annotators [2].

De-identification refers to the process of masking or replacing identifiable information. Here, identifiable information is defined as Protected Health Information (PHI), see Health Insurance Portability and Accountability (HIPAA), [3]. The de-identification task is very similar to the Named Entity Recognition (NER) task, which has been successfully used in NLP and IA systems. An example on using NER for de-identification of clinical text written in Swedish is described in [4]. There exist quite a few resources that have been annotated for NER purposes, such as for the MUC conferences (Message Understanding Conferences), [5] However, as pointed out by [6], the fundamental question of defining which annotations such systems should be able to handle, and how the annotators interpret these definitions, is often not addressed. For de-identification, defining identifiable instances and their scope is a very important issue. In Additional file 1 (Table S1), an overview of the annotation classes used for de-identification tasks are shown. As can be seen, several different ways of defining identifiable instances in EPRs have been employed by different research groups.

Moreover, de-identifying clinical corpora pose specific problems, as such corpora have properties that differ from other types of text, mainly in grammaticality and in levels of noise [7] describes work on annotating clinical corpora for Named Entities. Although the work is not intended for the purpose of de-identification, similarities in the annotation task for such language use is presented. For instance, problematic aspects such as variants in the representation of entities are discussed.

Automatic de-identification systems are mainly of two types: rule- and dictionary based or based on machine learning algorithms. There exist many different de-identifiers developed for English clinical text, for example, rule-based systems such as the Scrub system [8], the de-identification software engine by Gupta et al. [9], and De-id described in [10]. De-id is evaluated on a gold standard of 1 836 nursing notes containing 300 000 tokens. For other languages, rule based de-identification systems have also been developed, for instance Medina for French [11] and the Kokkinakis and Thurin system [4] for Swedish. Statistical or machine learning based de-identification systems for English include Stat De-id [12]. Seven de-identification systems (including one rule-based system and Stat De-id) are described in [13]. These systems are used in the first i2b2 challenge which consists of 889 discharge letters containing 470 000 tokens for training and 140 000 tokens for testing respectively. The training corpus contained 14 000 annotation instances distributed over eight annotation classes. One of the highest performing systems in [13] used the machine learning algorithm Conditional Random Fields (CRF), obtaining an F-score > 0.95.

Different machine learning algorithms are better suited for different classification problems. In both [13] and [14], Conditional Random Fields (CRF), and Support Vector Machines (SVM) are compared for the task of classifying entities in clinical text. In [14] both algorithms are applied on a small subset of clinical text written in English. The algorithms were trained on 1 265 annotations and evaluated on 292 annotations. The results show that CRF outperforms SVM for these types of classification tasks, producing an F-score of 0.86 and 0.64 respectively.

However, all systems mentioned above use resources that are annotated with different annotation classes, in many cases with different granularity (see Additional file 1, Table S1), and results from the different de-identification systems are therefore difficult to compare. Moreover, the resources are gathered from different types of clinical corpora (discharge letters, pathology reports, etc.), and both language use and number of identifiable instances may differ greatly, which makes results even more difficult to compare across systems. Also, portability to other languages is difficult to ensure, as language differences may affect system performance.

## Methods

### Refinement of a Gold Standard

We have previously started the process of creating a Gold standard for de-identification of the Stockholm EPR Corpus (Appendix). Three annotators annotated 100 patient records containing both free text and structured information, encompassing a total of 380 000 tokens. Identifiable instances were defined for the 18 Protected Health Information (PHI) classes given in [3] with some changes. In total 40 annotation classes were defined, including four nested classes and some additional classes, however only 28 of the defined 40 annotation classes were used for annotation, (see Additional file 2, Table S2 for the used 28 classes).

The creation of the Gold standard, the annotation guidelines and the resulting set of annotation classes is described in [15].

The average Inter-Annotator Agreement (IAA) result for all instances of the annotation classes on the Gold standard was 0.65 F-score. Some classes showed higher agreement than others, and the total number of annotations differed between the annotators. The approach taken for the creation of the Gold standard was deliberately coarse and loosely defined, for the purpose of getting an initial idea of what type of identifiable instances the EPRs actually contain. The Gold standard has been further analysed in the work presented here, and used for the creation of two refined consensus sets.

### Automatic Consensus Gold Standard

Our first approach to refine the Gold standard was to automatically create a union of all three annotation sets. One requirement for evaluating a de-identification system is that high recall is preferable over high precision, therefore we took the union of all annotations. Whenever there was a mismatch found, majority decision was prioritized. If two annotations covered almost the same instance, the longest instance span was chosen.

Moreover, as many classes were mismatched, a semi-automatic decision on resolving these discrepancies was made (if it could not be resolved by majority decision). For example, if an instance was annotated only by two annotators, and one annotator annotated the instance as *Clinician_First_Name *and the other as *First_Name*, the instance was annotated as *Clinician_First_Name*. Rules for resolving such cases were written after analyzing common mismatches for all annotation classes. All instances that were annotated only by one annotator were also included in the final set of annotation instances. This process resulted in a total amount of 6 170 annotation instances.

As many of the annotation classes are conceptually similar, several variants of merging similar (and removing some infrequent) classes were also made. This was done in order to evaluate whether the automatic classifier would perform better on more general, merged annotation classes.

### Manual Consensus Gold Standard

By creating pairwise matrices covering the total amount of annotations for each annotator, as well as an agreement table [16], covering all annotated instances and their number of assigned class judgments, a better overview of the class distributions, annotation instances and annotator judgements was obtained. In total, over 7 000 instances were annotated. However, the total amount of annotations per annotator could differ with over 1 000 instances. Many of these differences were due to boundary discrepancies and class mismatches.

In general, the distribution of annotation instances was very skewed. The annotation class *Health_Care_Unit *contained, by far, the largest amount of annotation instances. Some of the HIPAA classes were not present at all in the data set, such as *Social_Security_Number *and *Medical_Record_Number*. Only 28 of the defined 40 annotation classes were used for annotation. IAA was highest for the Name classes, see [15].

The analysis of the pairwise matrices and the agreement tables resulted in the identification of some differences in the interpretation of the guidelines. In particular, the use of the annotation class *Health_Care_Unit *differed greatly with a very low IAA, see [15]. These discrepancies were discussed jointly by the group of annotators and resulted in a more refined set of guidelines.

The main changes to the guidelines were the following:

• An instance should never be sub-tokenized by the annotator. For example, *34-årig *(Aged 34) should be annotated in its entirety.

• The *Relation *and *Ethnicity *classes were deleted. The annotators judged that these classes did not pose a high risk of identifying individual patients.

• The classes *Street_Address*, *Town*, *Municipality*, *Country *and *Organization *were merged into the more general class *Location*. Many of these classes were confused in the individual sets of annotations but covered the same instances. Moreover, the largest possible span should always be used for such instances. An address such as *Storgatan 1, 114 44 Stockholm *should be annotated in its entirety.

• Dates should never include weekdays. The division between *Date_Part *and *Full_Date *should be kept.

• Health care units should be annotated with the largest possible span, and should only be annotated if they denote a specific unit.

• General units that are not identifiable in themselves should not be annotated. A general unit such as *Geriatriken *(the Geriatrics department) should not be annotated if it was not specified by its hospital.

As stated above, the class *Health_Care_Unit *was the most problematic. In the EPRs, these instances could be mentioned in a variety of ways. Moreover, in the Stockholm area, many health care units have names that include their location. *Karolinska Universitetssjukhuset *(Karolinska University Hospital), for example, is located both in Huddinge and Solna, and the respective locations are included in their names. In the EPRs, these hospitals (and clinics within these hospitals) could be mentioned as for example:

Karolinska Univ. Sjukh, Huddinge

Karolinska/Huddinge

Avd. 11 på Karolinska

Moreover, in some cases, the hospital was referred to as *Karolinska i Solna *(Karolinska in Solna), where *Solna *in this case denotes a *Location*. Following the new guidelines, the longest span possible should always cover the instance, but only if the referred unit was specific. The definition of a general unit has, however, not been specified in detail but is left to be judged by the annotators. Such instances may still be a source of error.

A new, refined Gold standard was created semi-automatically after resolving these differences. Many annotations in the initial Gold standard did not conform to the new guidelines (weekdays annotated as *Date_Part *and generic health care units for instance) and were deleted. This resulted in a total amount of 4 423 annotation instances.

### Using the Consensus Gold Standards with a CRF Classifier

We have used the two created Consensus Gold standards to train and evaluate a Conditional Random Fields (CRF) classifier. As discussed above, such classifiers have shown promising results for de-identification classification tasks.

We have used the Stanford Named Entity Recognizer [17] using the default settings for all experiments.

All experiments have been evaluated with four-fold cross-validation [18] where the total set has been split into four equally sized sub-sets used for training and evaluation. The reason to use four-fold cross validation for the evaluation was to have a reasonable processing time.

Seven experiments using the automatic Consensus Gold standard are reported, each with different mergings of the annotation classes into more general classes and two experiments using the manual Consensus Gold standard, one evaluated with ten-fold cross-validation. No nested annotation classes were used.

## Results

### Using the Automatic Consensus Gold Standard

In the initial experiment, all 28 annotation classes are used in the classifier (see Additional file 2, Table S2). Some annotation classes contained very few annotations. Four-fold cross-validation on the 28 annotation classes, 6 170 annotation instances and 380 000 tokens in total took around 8 hours to execute on a server with Dual CPU Quad Core Intel Xeon E5405, 2.0 GHz with total 8 kernels and 16 Gb RAM.

By consecutively merging conceptually similar annotation classes, we tried to examine whether the classifier would increase the recall results as well as the overall performance. In the final experiments, all annotation instances are merged into one general PHI (Protected Health Information) class. In Additional file 3, Table S3 we see that, for all experiments, precision is very high when looking at the results for both exact and partial matches. An exact match is when the de-identification system finds exactly the same instance as the annotated data (token-level), a partial match is when the de-identification system matches partially on a character level. Recall, on the other hand, is much lower for exact matches than for partial matches. For de-identification purposes high recall is preferable over high precision, since it is more important to ensure a minimal risk of identification possibilities rather than ensuring trustworthiness of identified instances. Merging all annotation classes into one, general PHI class gives the highest F-score for partial matches. However, for exact matches, experiments 3 and 4 (using 16 or 13 annotation classes, respectively) give the best results.

It seems that the drop in performance for exact matches between experiment 4 and 5 mainly originates in a heavy overgeneration of names, where *First_Name *and *Last_Name *are grouped in the more generic class Name. However, looking at partial matches, the drop is not as dramatic, which indicates that there is some boundary problem here which might be due to initials or titles.

One conclusion is that conceptually similar annotation classes can be merged successfully, but not into too general classes. The amount of training instances for each class naturally affect results.

### Using the Manual Consensus Gold Standard

The manual Consensus Gold standard contained fewer annotation instances. When using this set in the CRF classifier, we merged all name classes into the generic *First_Name *and *Last_Name *respectively. We also merged *Age *and *Age_Over_89 *into one generic *Age*class. The annotation classes *Full_Date, Date_Part, Health_Care_Unit, Location*, and *Phone_Number *were also used. In Additional file 4, Table S4, the results on using this set are given. We see that the overall results are similar to the results on using the automatic Consensus Gold standard. However, given the smaller total amount of annotation instances, these results may be interpreted as being a bit better. In particular, the classes *Date_Part *and *Phone_Number *show much better results on the manual Consensus Gold standard (compare with Additional file 2, Table S2). The results for the classes *Health_Care_Unit *and *Location *are, for all experiments, relatively low. This is probably due to the ambiguous nature of many of the instances (i.e. *Huddinge *as a *Location *or *Health_Care_Unit*). Moreover, as can be seen in Additional file 1, Table S1, the initial classes *Street_Address*, *Town*, *Municipality*, and *Organization*, that have been merged in the manual Consensus Gold standard had few instances respectively. With more training instances, these results might improve. In Additional file 5, Table S5 we see the results on evaluating the CRF classifier (using the manual Consensus Gold standard) also with ten-fold cross-evaluation. We used ten-fold cross-evaluation to test whether more training data would improve our results. It is clear that the overall results for most classes improve considerably when providing more training data.

## Discussion

As stated above, it is difficult to compare these results to previous research due to differences in corpora, annotation classes, evaluation methods and also language. In particular, defining the appropriate set of annotation classes for de-identification tasks is challenging. In Additional file 6, Table S6 we can see the original set of annotation classes from [15], the used annotation classes, and finally the ones proposed after discussion among the annotators. However, given the small size of the corpus, we believe that our results are very promising. The lower results for the *Location *and *Health_Care_Units *classes can be compared with the results for the competing systems described in Uzuner et al. [12], where the results for these classes are consistently lower for all systems. Also, the generally high results for classes covering patients and clinicians can be compared to our high results on the name classes.

Notable are the general results for exact and partial matches. Naturally, the overall results for exact matches are generally lower, but the differences are not as drastic for the Manual Consensus Gold standard. This indicates that boundaries are difficult to identify for de-identification instances, which was also concluded during the discussions among the annotators, especially for the *Health_Care_Unit *class, and dealt with for the creation of this refined set.

When using manually annotated resources for training and evaluation, it is also interesting to scrutinize the resulting false positives from the classifier. In the experiment using the manual Consensus Gold standard, the Stanford NER also discovered in total 178 false positives, where 49 were actual true positives from the annotation classes *First_Name*, *Last_Name*, *Location*, *Health_Care_Unit*, *Date_Part *and *Full_Date*. Clearly, the human annotators missed out on identifiable information.

The automatic consensus took around one and half working week of implementation including some manual work and the manual consensus took around two and a half weeks of work including some programming. The advantage of the manual consensus creation was having control over the full process while in the automatic consensus previous errors and discrepancies were not handled.

To improve our results and minimize the efforts of manual annotation, we plan to use active learning [19], i.e., employ learning methods that not only generate predictive models from a given set of training examples, but also may suggest additional useful training examples. In this active learning scenario, the aim will be that the learning method requests a minimum set of extra annotated material to achieve sufficiently high performance.

## Conclusions

Fully de-identified EPR corpora are very important resources for the research community. With these, development of new methods for exploiting and exploring the valuable information contained in such data sets is possible. Moreover, it enables researchers to compare and evaluate findings in a more reliable manner. We have refined an existing de-identification Gold standard into two Consensus Gold standards. The refined Consensus Gold standards have been used in a CRF classifier with promising results. The automatic Consensus Gold standard has resulted in a larger set of annotation instances, where discrepancies have been resolved semi-automatically. The creation of this set required less cost in time, but contains more noise.

The manual Consensus Gold standard (The Stockholm EPR PHI Corpus), is the result of discussions within the group of annotators, and a new set of guidelines has been developed for future similar annotation tasks. At the end, the group of annotators settled for using the following set of annotation classes in the future: *Age, First_Name, Last_Name *(these are further refined for *Patient*, *Clinician *and *Relative*), *Date_Part, Full_Date, Location Health_Care_Unit, Phone_Number, E-mail_address *and *Social_Security_Number*. This set of annotation classes has passed through two iterations of thorough reviews, and our intention is to make this set available for a broader group of researchers in the future.

By merging conceptually similar annotation classes, it is possible to automatically refine an existing Gold standard with somewhat inconsistent annotations and improve results, but better results, both for exact and partial matches, are obtained by systematically identifying inconsistencies (through analysis and discussions) and refining the annotations thereafter. For this work, we conclude that, despite the slightly more costly procedure of refining an existing set of de-identification annotations manually, the resulting set is more reliable for further research.

However, as the size of the corpus is relatively small, and the amount of instances for some annotation classes is very low, more training material would be needed in order to produce more stable results. Some annotation classes such as social security number and patient names will probably be very scarce in the EPRs. We will therefore need other approaches to capture these annotation instances. One possibility is to use a rule- and dictionary based method for de-identification of such instances.

In our experiments with CRF we have used the default settings of Stanford CRF for all experiments, which, in this case, meant using distributional feature sets with n-grams up to size six. Further analysis on and evaluation of useful and extended features as well as weighting schemes for this specific classification problem is needed.

Defining annotation classes for de-identification is difficult. Moreover, EPRs use a language which is very noisy and rich in variations of expressions. Such properties makes clear definitions on boundaries and coverage of annotation classes challenging. We have further outlined the criteria needed for the creation of an annotated EPR corpus for de-identification, but many questions may still arise in the future.

We believe that the resulting set of annotation classes obtained after discussions is useful for similar tasks, as it covers the most important identifiable instances. However, even if it would be possible to guarantee optimal performance for these classes, it is impossible to ensure that no individual patient can be identified from the information remaining in an EPR.

## Competing interests

The authors declare that they have no competing interests.

## Authors' contributions

HD planned the research work, set up the experiments and wrote the paper. SV did the consensus work, ran the CRF system and wrote the paper. All authors read and approved the final manuscript.

## Appendix

This research has been carried out after approval from the Regional Ethical Review Board in Stockholm (Etikprövningsnämnden i Stockholm), permission number 2007/1625-31/5.

## Supplementary Material

Additional file 1 **An overview of annotation classes**. An overview of annotation classes used for de-identification by different research groups on clinical corpora. Click here for file

Additional file 2 **(Table 2) Results of the initial experiment using all 28 classes from the automatic Consensus Gold standard**. Results of the initial experiment using all 28 classes from the automatic Consensus Gold standard, giving results on exact matches. The different divisions show which classes have been merged for the remaining six experiments on the automatic Consensus Gold standard. The classes Person name and Location have been further collapsed from the original sets of classes. The column Annotated contains the number of annotated classified instances (Gold Standard). The column Retrieved contains the number of retrieved instances (produced by the CRF classifier). The column *Relevant* contains the number of correctly retrieved instances. Click here for file

Additional file 3 **Results of all seven experiments using the automatic Consensus Gold standard**. The total average over all classes is given. For each new experiment, conceptually similar annotation classes are merged into a more general annotation class. The final experiment shows the results of merging all annotation classes into one general PHI class. Click here for file

Additional file 4 **Results of the manual Consensus Gold standard using four-fold cross-evaluation**. Click here for file

Additional file 5 **Results of the manual Consensus Gold standard using ten-fold cross-evaluation**. Click here for file

Additional file 6 **Initial annotation classes, used annotation classes and proposed annotation classes**. *Initial annotation classes *are those proposed in [15]. *Used annotation classes *are those that were used in the creation of the first Gold Standard (100 EPRs), also described in [15]. *Proposed annotation classes *are the ones proposed in this article, which arose from consensus discussions among the annotators. Click here for file
