# Supplementary material for: De-identifying Swedish clinical text - refinement of a gold standard and experiments with Conditional random fields
Source: J Biomed Semantics. 2010 Apr 12;1:6. doi: 10.1186/2041-1480-1-6 (PMC2895734; doi:10.1186/2041-1480-1-6)
Supplement: Additional file 1 — An overview of annotation classes. An overview of annotation classes used for de-identification by different research groups on clinical corpora. [file 2041-1480-1-6-S1.PDF]

No	1	2	3	4	5	6	7	8	9	10	11	12	13	14	15	16	17	18	19	20	21	22	23	24	25	26	27	28
**Annotation classes**	First Name	Last Name	Name	Clinician Name	Proxy/Relative Name	Patient Name	Patient Name initial	Hospital	Disease	Pharma/ names	Measure	Organization	Adress	Location	Employer	Job title	Phone Number	e-mail address	Social security number	Medical record number	ID	Account number	Date	Year	Age	Age over 89	Ethinicty/ Holiday	Undefined
Gupta et al. 2004			X		X								X		X	X	X	X	X	X		X	X					
Uzuner et al. 2007				X		X		X						X			X	X			X		X					
Kokkinakis & Thurin 2007 (Swedish)			X						X	X	X	X		X									X					
Neamatullah et al. 2008				X	X	X	X							X			X							X	X	X	X	X
Grouin et al 2009 (French)	X	X												X									X		X			

Additional file 1 (Table S1) – An overview of annotation classes

An overview of annotation classes used for de-identification by different research groups on clinical corpora
