# Supplementary material for: De-identifying Swedish clinical text - refinement of a gold standard and experiments with Conditional random fields
Source: J Biomed Semantics. 2010 Apr 12;1:6. doi: 10.1186/2041-1480-1-6 (PMC2895734; doi:10.1186/2041-1480-1-6)
Supplement: Additional file 2 — (Table 2) Results of the initial experiment using all 28 classes from the automatic Consensus Gold standard. Results of the initial experiment using all 28 classes from the automatic Consensus Gold standard, giving results on exact matches. The different divisions show which classes have been merged for the remaining six experiments on the automatic Consensus Gold standard. The classes Person name and Location have been further collapsed from the original sets of classes. The column Annotated contains the number of annotated classified instances (Gold Standard). The column Retrieved contains the number of retrieved instances (produced by the CRF classifier). The column Relevant contains the number of correctly retrieved instances. [file 2041-1480-1-6-S2.PDF]

	Exact matches<br>Class	Annotated	Retrieved	Relevant	Precision	Recall	F-score
	Account_Number	1	0	0	NaN	0.000000	NaN
**AGE**	Age	54	45	40	0.888889	0.740741	0.808081
	Age_Over_89	3	0	0	NaN	0.000000	NaN
	Biometric_Identifier	4	0	0	NaN	0.000000	NaN
**DATE**	Date_Part	843	682	629	0.922287	0.746145	0.824918
	Full_Date	503	427	338	0.791569	0.671968	0.726882
	Year	62	22	15	0.681818	0.241935	0.357143
**PERSON NAME**	First_Name	13	0	0	NaN	0.000000	NaN
	Last_Name	5	0	0	NaN	0.000000	NaN
	Patient_First_Name	75	14	6	0.428571	0.080000	0.134831
	Patient_Last_Name	3	0	0	NaN	0.000000	NaN
	Relative_First_Name	128	72	67	0.930556	0.523438	0.670000
	Relative_Last_Name	23	8	8	1.000000	0.347826	0.516129
	Clinician_First_Name	735	612	541	0.883987	0.736054	0.803267
	Clinician_Last_Name	901	764	706	0.924084	0.783574	0.848048
**LOCA-TION**	Location	3	0	0	NaN	0.000000	NaN
	Country	27	7	5	0.714286	0.185185	0.294118
	Municipality	34	14	3	0.214286	0.088235	0.125000
	Organization	60	1	0	0.000000	0.000000	NaN
	Street_Address	12	0	0	NaN	0.000000	NaN
	Town	52	12	7	0.583333	0.134615	0.218750
	Health_Care_Unit	1747	1162	910	0.783133	0.520893	0.625645
	Device_Identifier_and_Serial_Numbe	6	0	0	NaN	0.000000	NaN
	Ethnicity	9	0	0	NaN	0.000000	NaN
**PHONE-NUMBER**	Fax_Number	5	0	0	NaN	0.000000	NaN
	Phone_Number	130	65	56	0.861538	0.430769	0.574359
	Relation	714	473	458	0.968288	0.641457	0.771693
	Uncertain	18	1	0	0.000000	0.000000	NaN
	Total	6170	4381	3789	0.864871	0.614100	0.718226

Additional file 2 (Table S2) - Results of the initial experiment using all 28 classes from the automatic Consensus Gold standard

Results of the initial experiment using all 28 classes from the automatic Consensus Gold standard, giving results on exact matches. The different divisions show which classes have been merged for the remaining six experiments on the automatic Consensus Gold standard. The classes Person name and Location have been further collapsed from the original sets of classes. The column *Annotated* contains the number of annotated classified instances (Gold Standard). The column *Retrieved* contains the number of retrieved instances (produced by the CRF classifier). The column *Relevant* contains the number of correctly retrieved instances.
