# Supplementary material for: De-identifying Swedish clinical text - refinement of a gold standard and experiments with Conditional random fields
Source: J Biomed Semantics. 2010 Apr 12;1:6. doi: 10.1186/2041-1480-1-6 (PMC2895734; doi:10.1186/2041-1480-1-6)
Supplement: Additional file 3 — Results of all seven experiments using the automatic Consensus Gold standard. The total average over all classes is given. For each new experiment, conceptually similar annotation classes are merged into a more general annotation class. The final experiment shows the results of merging all annotation classes into one general PHI class. [file 2041-1480-1-6-S3.PDF]

					Exact matching			Partial matching		
**Experiment**	**Annotation classes**	**Annotated**	**Retrieved**	**Relevant**	**Precision**	**Recall**	**F-score**	**Precision**	**Recall**	**F-score**
1	28	6170	4381	3789	0.864871	0.614100	0.718226	0.910709	0.645112	0.755240
2	22	6171	4439	3910	0.880829	0.633609	0.737041	0.925873	0.664288	0.773565
3	16	6160	4501	4013	0.891580	0.651461	0.752837	0.939449	0.683472	0.791273
4	13	6135	4498	4016	**0.892841**	**0.654605**	**0.755384**	0.940548	0.686649	0.793790
5	9	5654	4050	3147	0.777037	0.556597	0.648599	0.933757	0.673440	0.782517
6	7	5642	4053	3150	0.777202	0.558313	0.649819	0.933730	0.675193	0.783689
7	1	5453	4177	3040	0.727795	0.557491	0.631360	**0.937036**	**0.714620**	**0.810852**

Additional file 3 (Table S3) - Results of all seven experiments using the automatic Consensus Gold standard

The total average over all classes is given. For each new experiment, conceptually similar annotation classes are merged into a more general annotation class. The final experiment shows the results of merging all annotation classes into one general PHI class.
