# Supplementary material for: De-identifying Swedish clinical text - refinement of a gold standard and experiments with Conditional random fields
Source: J Biomed Semantics. 2010 Apr 12;1:6. doi: 10.1186/2041-1480-1-6 (PMC2895734; doi:10.1186/2041-1480-1-6)
Supplement: Additional file 4 — Results of the manual Consensus Gold standard using four-fold cross-evaluation. [file 2041-1480-1-6-S4.PDF]

				Exact matches			Partial matches		
**Class**	**Annotated**	**Retrieved**	**Relevant**	**Precision**	**Recall**	**F-score**	**Precision**	**Recall**	**F-score**
**Age**	56	41	37	0.902439	0.660714	0.762887	0.947735	0.719388	0.817923
**Date_Part**	710	643	605	0.940902	0.852113	0.894309	0.943721	0.854829	0.897078
**Full_Date**	500	427	332	0.777518	0.664000	0.716289	**0.922879**	**0.791425**	**0.852112**
**First_Name**	923	718	676	0.941504	0.732394	0.823888	0.944290	0.733686	0.825771
**Last_Name**	929	757	715	**0.944518**	**0.769645**	**0.848161**	0.953967	0.776767	0.856296
**Health_Care_Unit**	1021	594	455	0.765993	0.445642	0.563467	0.903736	0.499340	0.643260
**Location**	148	65	44	0.676923	0.297297	0.413146	0.720513	0.311562	0.435015
**Phone_Number**	136	70	65	0.928571	0.477941	0.631068	0.949649	0.490950	0.647273
**Total**	4423	3315	2929	0.883560	0.662220	0.757043	0.932133	0.692842	0.794869

Additional file 4 (Table S4) - Results of the manual Consensus Gold standard using four-fold cross-evaluation
