# Supplementary material for: De-identifying Swedish clinical text - refinement of a gold standard and experiments with Conditional random fields
Source: J Biomed Semantics. 2010 Apr 12;1:6. doi: 10.1186/2041-1480-1-6 (PMC2895734; doi:10.1186/2041-1480-1-6)
Supplement: Additional file 5 — Results of the manual Consensus Gold standard using ten-fold cross-evaluation. [file 2041-1480-1-6-S5.PDF]

				Exact matches			Partial matches		
**Class**	**Annotated**	**Retrieved**	**Relevant**	**Precision**	**Recall**	**F-score**	**Precision**	**Recall**	**F-score**
**Age**	56	45	37	0.822222	0.660714	0.732673	0.904762	0.778061	0.836642
**Date_Part**	710	654	617	0.943425	0.869014	0.904692	0.946196	0.871730	0.907438
**Full_Date**	500	426	342	0.802817	0.684000	0.738661	**0.931665**	**0.802106**	**0.862045**
**First_Name**	923	749	713	0.951936	0.772481	0.852871	0.954606	0.773772	0.854729
**Last_Name**	928	816	777	**0.952206**	**0.837284**	**0.891055**	0.961653	0.845484	0.899835
**Health_Care_Unit**	1021	689	559	0.811321	0.547502	0.653801	0.921497	0.608116	0.732705
**Location**	148	73	54	0.739726	0.364865	0.488688	0.778539	0.379129	0.509933
**Phone_Number**	135	86	80	0.930233	0.592593	0.723982	0.954195	0.613105	0.746535
**Total**	4421	3538	3179	0.898530	0.719068	0.798844	0.941190	0.751441	0.835680

Additional file 5 (Table S5) - Results of the manual Consensus Gold standard using ten-fold cross-evaluation
