# Supplementary material for: De-identifying Swedish clinical text - refinement of a gold standard and experiments with Conditional random fields
Source: J Biomed Semantics. 2010 Apr 12;1:6. doi: 10.1186/2041-1480-1-6 (PMC2895734; doi:10.1186/2041-1480-1-6)
Supplement: Additional file 6 — Initial annotation classes, used annotation classes and proposed annotation classes. Initial annotation classes are those proposed in [15]. Used annotation classes are those that were used in the creation of the first Gold Standard (100 EPRs), also described in [15]. Proposed annotation classes are the ones proposed in this article, which arose from consensus discussions among the annotators. [file 2041-1480-1-6-S6.PDF]

Initial annotation classes inspired from HIPAA	Used annotation classes	Proposed annotation classes
Account_Number	Account_Number	
**Age**<br>**Age_Over_89**	**Age**<br>**Age_Over_89**	**Age**
Biometric_Identifier	Biometric_Identifier	
Social_security_number		
Medical_record_number		
Health_plan_beneficiary_number		
Certificate_or_license_number		
Vehicle_identifier		
Device_identifiers_and_serial_number	Device_Identifier_and_Serial_Number	
**Full_Date**	**Full_Date**	**Full_Date**
**Date_Part**<br>**Year**	**Date_Part**<br>**Year**	**Date_Part**
Full_Name		**First_Name**<br>**Last_Name**
**First_Name**<br>**Last_Name**	**First_Name**<br>**Last_Name**	
Patient_Full_Name		
**Patient_First_Name**<br>**Patient_Last_Name**	**Patient_First_Name**<br>**Patient_Last_Name**	
Relative_Full_Name		
**Relative_First_Name**<br>**Relative_Last_Name**	**Relative_First_Name**<br>**Relative_Last_Name**	
Clinician_Full_Name		
**Clinician_First_Name**<br>**Clinician_Last_Name**	**Clinician_First_Name**<br>**Clinician_Last_Name**	
**Location**<br>**Country**<br>**Municipality**<br>**Organization**<br>**Street_Address**<br>**Town**	**Location**<br>**Country**<br>**Municipality**<br>**Organization**<br>**Street_Address**<br>**Town**	**Location**
**Health_Care_Unit**	**Health_Care_Unit**	**Health_Care_Unit**
Ethnicity	Ethnicity	
**Fax_Number**<br>**Phone_Number**	**Fax_Number**<br>**Phone_Number**	**Phone_Number**
E-mail_address		
Web_URL		
IP_address_number		
Relation	Relation	
Uncertain	Uncertain	

Additional file 6 (Table S6) – Initial annotation classes, used annotation classes and proposed annotation classes.

Initial annotation classes are those proposed in [15]. *Used annotation classes* are those that were used in the creation of the first Gold Standard (100 EPRs), also described in [15]. *Proposed annotation* classes are the ones proposed in this article, which arose from consensus discussions among the annotators.
